# About Plastic Stoppings

**Published:** 1884-08

**Authors:** 


### ABOUT PLASTIC STOPPINGS.

At a recent meeting of the New York Odontological Society, Dr. F. Y. Clark spoke of the combination of amalgam with oxy-phosphate of zinc as a filling material, and gave his method of preparing the mass.

The suggestion, emanating from so creditable a source, and the idea seeming so sensible, prompted the query as to whether such a combination might not serve a better purpose in many cases than either of these materials used separately?

The principal objection to alloy is its tendency to change shape, and so to leave the cavity-margins insecure, and zinc fillings too rapidly wear or wash away to be of permanent value. In the combination referred to these objections are much modified, and repeated experiments recently made give a favorable impression of its value.

The proportions stated were one part amalgam to two parts of the zinc. The former is prepared as for an ordinary filling, and when the cavity is ready for receiving the mass, should be well rubbed with a spatula into the soft paste of the zinc phosphate. It should not be mixed too stiff, especially where cavities are large and the walls frail. Sufficient allowance should also be made in finishing for veneering the exposed surface with a light coating of amalgam, which will readily adhere to the stopping.

When properly mixed it is exceedingly sticky, and clings tenaciously to whatever it touches. It is excellent for securing gold crowns, and where the inside of the caps are slightly "amalgamated" they will adhere firmly to the mass. Care, however, should be taken to avoid getting mercury on the outer surface of the gold.

Cases are presented in the course of a dentist's experience where this combination may be used to advantage, and Dr. Clark is entitled to thanks for his suggestion. C. E. F.

